# Supplementary material for: Meta-analysis shows the impacts of ecological restoration on greenhouse gas emissions
Source: Nat Commun. 2024 Mar 26;15:2668. doi: 10.1038/s41467-024-46991-5 (PMC10965928; doi:10.1038/s41467-024-46991-5)
Supplement: Supplementary file 1 — Supplementary Information [file 41467_2024_46991_MOESM1_ESM.pdf]

Supplementary Information

Meta-analysis shows the impacts of ecological restoration on greenhouse gas emissions

Tiehu He[1,2,3,4], Weixin Ding[5], Xiaoli Cheng[6], Yanjiang Cai[7], Yulong Zhang[8], Huijuan Xia[1,2], Xia Wang[1,2], Jiehao Zhang[1,2], Kerong Zhang[1, 2, 3, 4*], Quanfa Zhang [1, 2]

1 Key Laboratory of Aquatic Botany and Watershed Ecology, Wuhan Botanical Garden, Chinese Academy of Sciences, Wuhan 430074, P.R. China

2 Danjiangkou Wetland Ecosystem Field Scientific Observation and Research Station, the Chinese Academy of Sciences & Hubei Province, Wuhan 430074, P.R. China

3 Key Laboratory of Lake and Watershed Science for Water Security, Nanjing Institute of Geography and Limnology, Chinese Academy of Sciences, Nanjing 210008, China

4 Hubei Key Laboratory of Wetland Evolution & Ecological Restoration, Wuhan Botanical Garden, Chinese Academy of Sciences, Wuhan 430074, China

5State Key Laboratory of Soil and Sustainable Agriculture, Institute of Soil Science, Chinese Academy of Sciences, Nanjing 210008, China

6 School of Ecology and Environmental Science, Yunnan University, Kunming 650091, P. R. China

7 State Key Laboratory of Subtropical Silviculture, Zhejiang A&F University, Hangzhou 311300, China

8 Eastern Forest Environmental Threat Assessment Center, Southern Research

Station, USDA Forest Service, Research Triangle Park, NC, 27709, USA

*Corresponding author: Kerong Zhang, Ph.D., Professor

Email: kerongzhang@wbgcas.cn

Key Laboratory of Aquatic Botany and Watershed Ecology, Wuhan Botanical Garden,

Chinese Academy of Sciences, Wuhan 430074, P. R. China

Phone: +86 27 87700866 Fax: +86 27 87510251

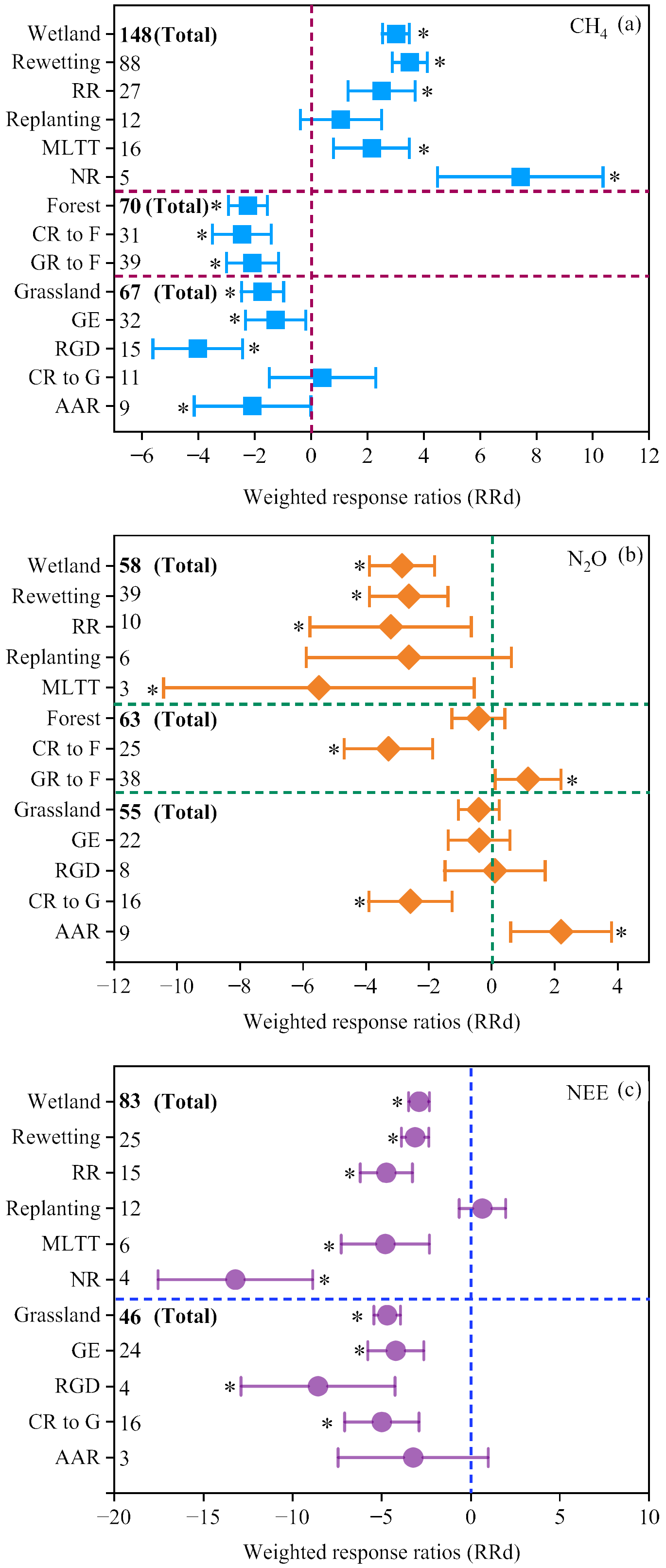

Supplementary Figure S1 Effects of ecological restoration measures on CH_4 (a), N_2O (b), and NEE (c) fluxes across the different wetland, forest and grassland restoration categories.

The overall effect size was calculated with a categorical random effects model. Values are means ±95% CIs of the weighted response ratios (*RRd*) between the paired control ecosystems and restored ecosystems. If the 95% CI value does not overlap with zero, the response is considered significant. The asterisks indicate significant positive or negative effects. Numbers next to the y-axis indicate sample sizes (n). MLTT, moss layer transfer technique; NR, naturally regeneration; RR, replanting and rewetting; CR to F, cropland to forest; GR to F; grassland to forest; GE, grazing exclusion; RGD, reduced grazing density; CR to G, cropland to grassland; AAR, artificial assisted restoration. Source data are provided as a Source Data file.

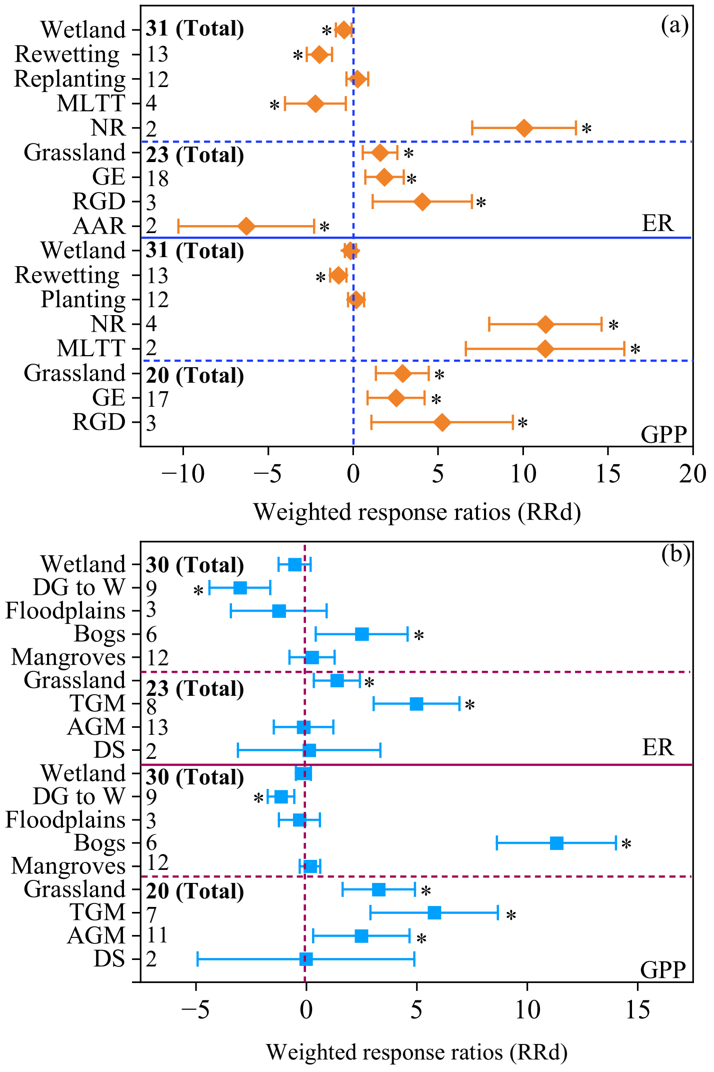

Supplementary Figure S2 Effects of ecological restoration measures (a) and types (b) on CO_2 (GPP and ER) fluxes across the different wetland, forest and grassland restoration categories.

The overall effect size was calculated with a categorical random effects model. Values are means ±95% CIs of the weighted response ratios (*RRd*) between the paired control ecosystems and restored ecosystems. If the 95% CI value does not overlap with zero, the response is considered significant. The asterisks indicate significant positive or negative effects. Numbers next to the y-axis indicate sample sizes (n). GPP, gross primary productivity; ER, ecosystem respiration; MLTT, moss layer transfer technique; NR, naturally regeneration; RR, replanting and rewetting; RGD, reduced grazing density; AAR, artificial assisted restoration; DG to W; drained grassland to wetland; TGM, Temperate steppe & meadow; AGM, Alpine steppe & meadow; DS, Desert

steppe. Source data are provided as a Source Data file.

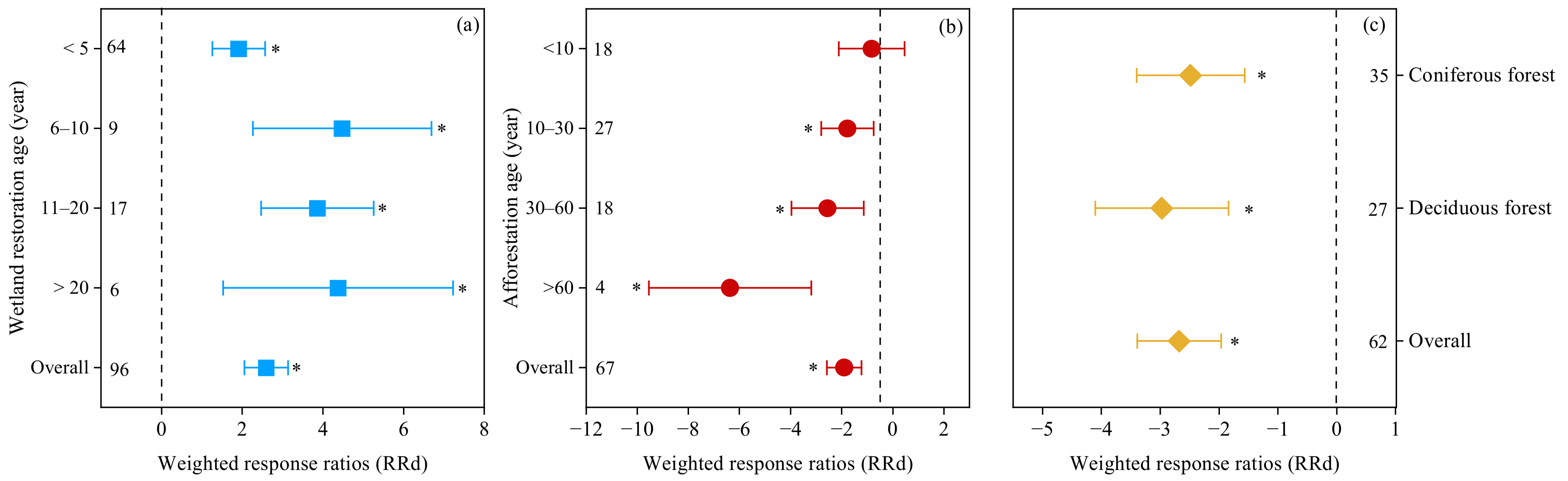

Supplementary Figure S3 The mean effect sizes of wetland restoration (a), afforestation (b), and tree species (c) on soil CH_4 emissions in different restoration age groups. The overall effect size was calculated with a categorical random effects model. Values are means ±95% CIs of the weighted response ratios (*RRd*) between the paired control ecosystems and restored ecosystems. If the 95% CI value does not overlap with zero, the response is considered significant. The asterisks indicate significant positive or negative effects. Numbers next to the y-axis indicate sample sizes (n). Source data are provided as a Source Data file.

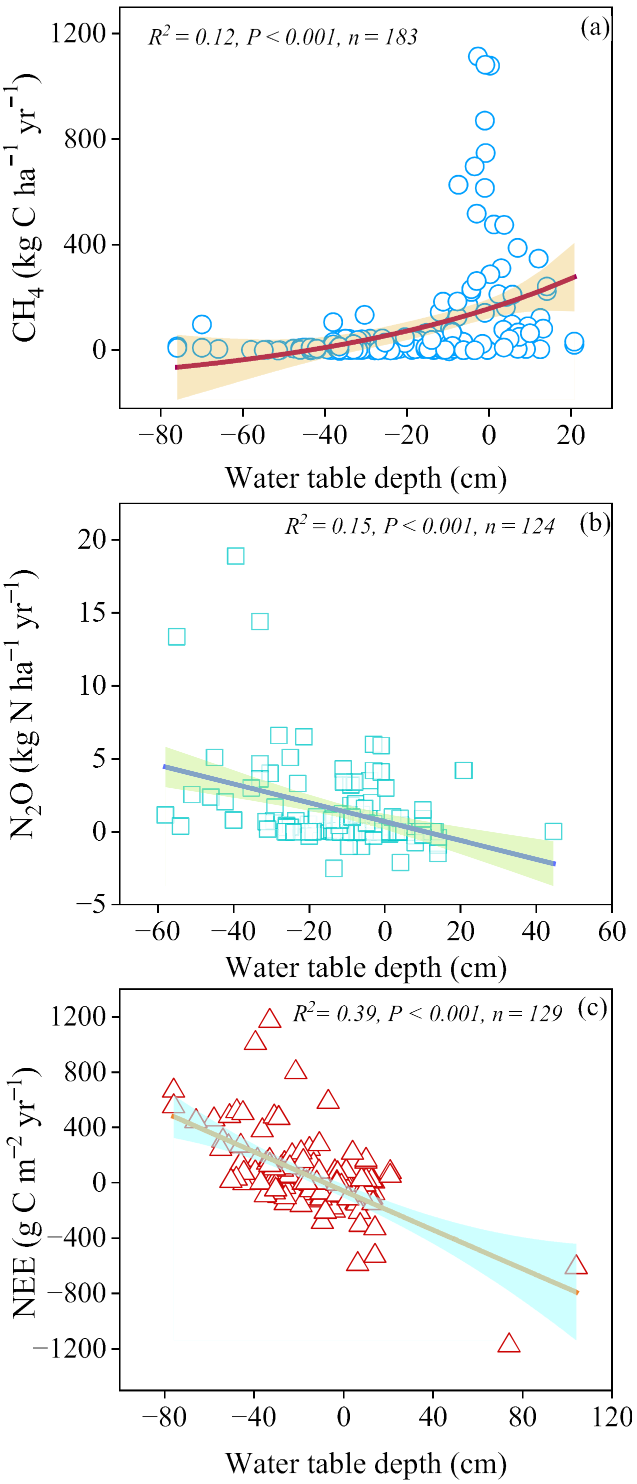

Supplementary Figure S4. Relationships of CH_4 emissions (a), N_2O emissions (b), and NEE (c) in restored wetlands with water table depth. Linear and nonlinear regression were used and the error bands surrounding the regression lines represent the 95% confidence interval of the correlation. Exact *p*-values and Source data are provided as a Source Data file.

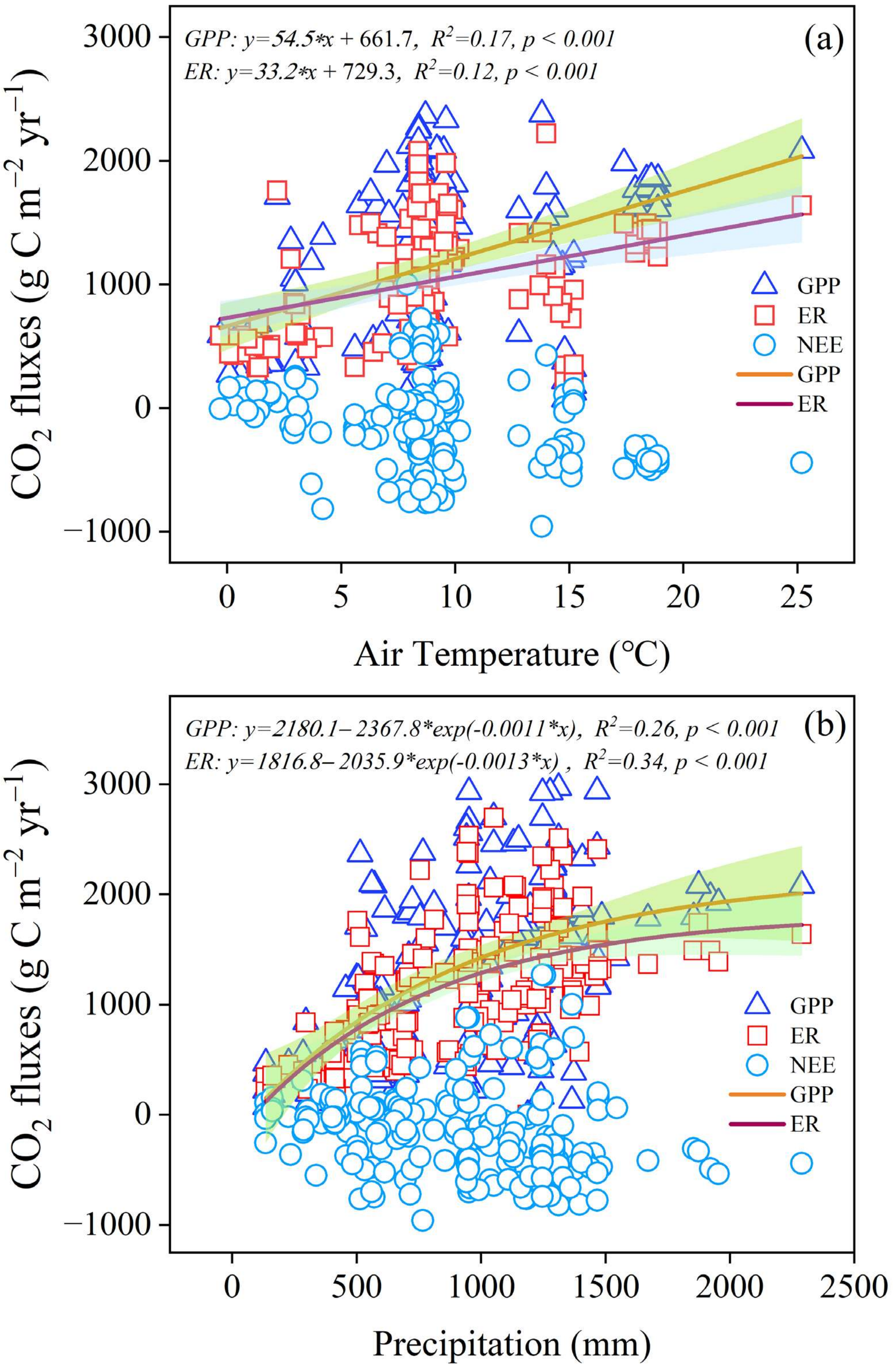

Supplementary Figure S5. Relationships of CO_2 fluxes with air temperature (a) and precipitation (b). Linear and nonlinear regression were used and the error bands surrounding the regression lines represent the 95% confidence interval of the correlation. Exact *p*-values and Source data are provided as a Source Data file.

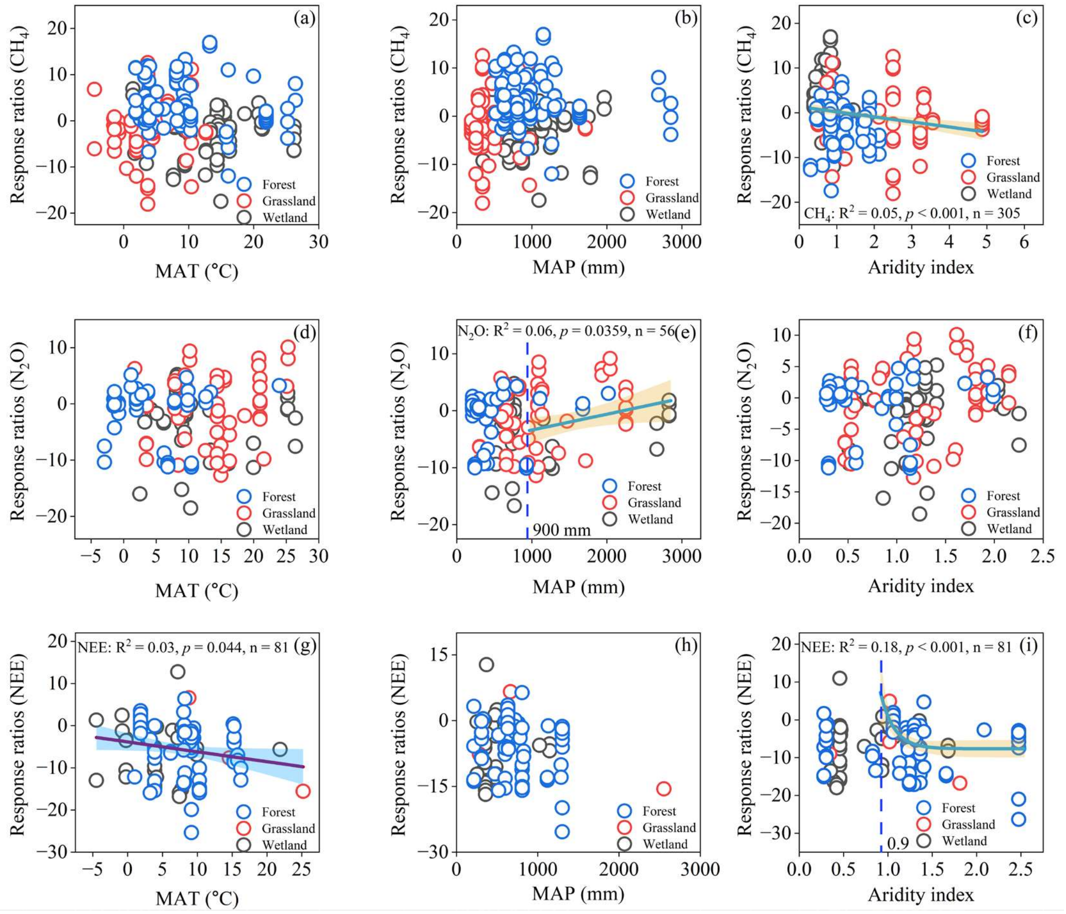

Supplementary Figure S6 Relationships of the response ratios (*RRd*) of CH_4 (a, b and c), N_2O (d, e and f), and NEE (g, h and i) with air temperature, precipitation, and aridity index. Linear and nonlinear regression were used and the error bands surrounding the regression lines represent the 95% confidence interval of the correlation. MAT, mean annual temperature; MAP, mean annual precipitation. The aridity index is the amount of average annual precipitation divided by the amount of potential evapotranspiration. The colored area around the regression line represents the 95% confidence interval, where n is the number of paired observations. Exact p-values and Source data are provided as a Source Data file.

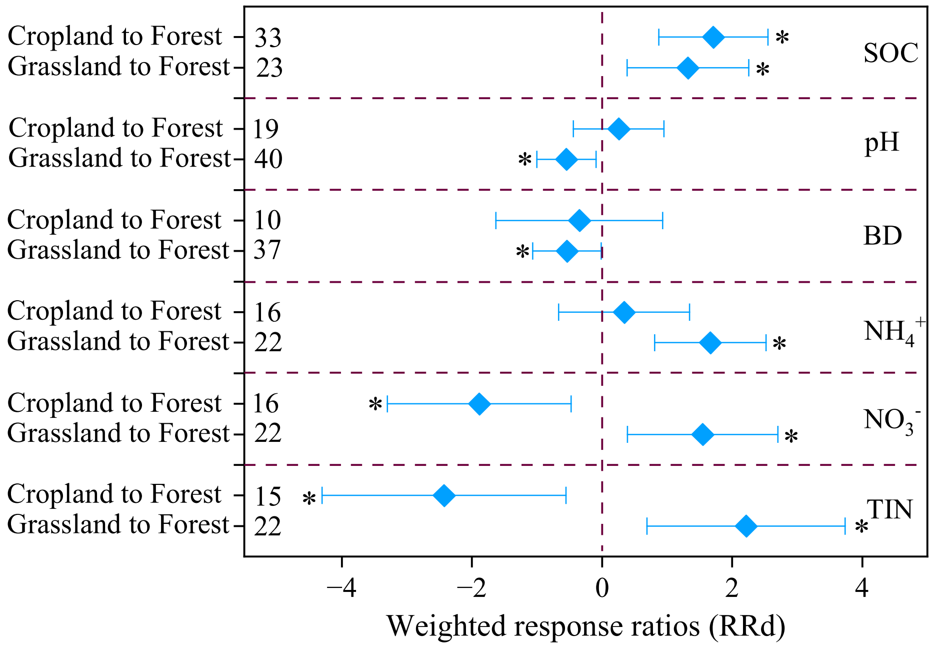

Supplementary Figure S7 Effects of the conversion of croplands and grasslands to forests on soil properties. The overall effect size was calculated with a categorical random effects model. If the 95% CI value does not overlap with zero, the response is considered significant. The asterisks indicate significant effects. The numbers next to the y-axis indicate sample sizes (n). SOC, soil organic carbon; BD, bulk density; TIN, the sum of NH_4^+ and NO_3^-. Values are means ±95% CIs of the weighted response ratios (*RRd*) between the paired control ecosystems and restored ecosystems. Source data are provided as a Source Data file.

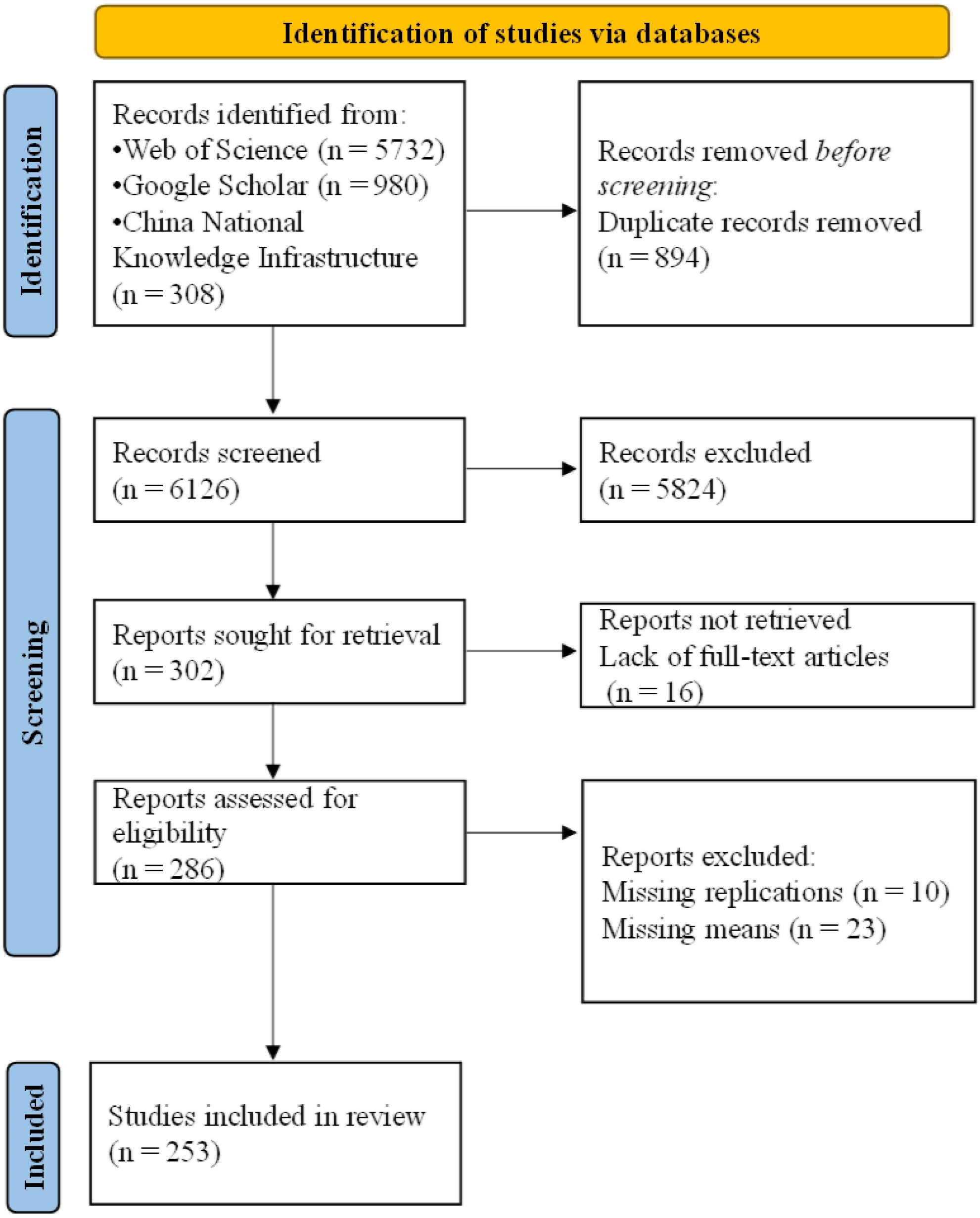

Supplementary Figure S8 A PRISMA flowchart showing the number of studies included and excluded at the literature search. For the full search string refer to Methods in the main text.

Table S1 Changes in comprehensive C budget and GWP when converting the paired control ecosystems (prefixed with 'P') to the restored wetland. ND, no data; NEE, net ecosystem CO_2 exchange; GWP, global warming potentials; C budget, the sum of NEE-C and CH_4-C. Source data are provided as a Source Data file.

Wetland Restoration	CH_4 kg C ha^{-1} $year^{-1}$	N_2O kg N ha^{-1} $year^{-1}$	NEE g C m^{-2} $year^{-1}$	C budget g C m^{-2} $year^{-1}$	GWP t CO_2-eq ha^{-1} $year^{-1}$	Rate of change %
P-Grasslands	61.2±13.6	5.2±1.4	231.9±93.8	238.0±95.2	12.9±4.5	
Wetlands	284.8±52.1	2.6±0.7	–219.5±62.9	–191.1±68.1	3.4±4.5	–73.8
P-Croplands	3.4±1.9	16.9±7.3	461±183.0	461.3±183.2	24.3±9.9	
Wetlands	182±46.1	2.3±0.6	–140.2±237.5	–121.9±242.1	2.4±10.6	–90.0
P-Forests	7.3±3.4	4.3±2.3	75.6±28.6	76.4±29.1	4.9±2.2	
Wetlands	39.7±11.25	2.2±2.1	10.5±58	14.4±59.6	2.8±3.6	–43.3
P-Aquaculture	225.1±135.1	ND	–41.9±0	–19.4±13.5	ND	
Wetlands	165.1±38.2	ND	–151.5±15.6	–135.0±19.4	ND	ND
P-Floodplains	–2.6±0.4	5.7±5.0	–166.7±84.5	–166.9±84.6	–3.7±5.3	
Restoration	42.8±39.7	3.1±1.9	–265.7±91.9	–261.4±95.8	–6.9±5.6	–82.9
P-Bogs	4.8±1.3	2.2±0.7	159.2±32.5	159.7±32.6	7.0±1.5	
Restoration	92.3±22.8	0.5±0.3	–35.8±25.2	–26.6±27.5	2.2±1.9	–68.0
P-Mangroves	198±0	8.4±2.8	147±0	166.8±0.0	16.2±1.2	
Restoration	215.5±6.5	4.3±1.3	164.8±10.4	186.4±11.1	15.7±1.2	–3.1
Total Control	23.4±6.9	6.7±1.6	176.5±42.1	178.8±42.7	10.2±2.5	
Total Restoration	150.8±17.1	2.1±0.3	–68.5±25.6	–53.4±27.3	3.9±1.7	–62.0

Table S2 Changes in comprehensive C budget and GWP by different ecological restoration measures. ND, no data; NEE, net ecosystem CO_2 exchange; C budget, the sum of NEE-C and CH_4-C; GWP, global warming potentials; MLTT, moss layer transfer technique; NR, naturally regeneration; RR, replanting and rewetting; RGD, reduced grazing density; GE, grazing exclusion; AAR, artificial assisted restoration. Source data are provided as a Source Data file.

Ecosystem	Restoration type	CH_4 kg C ha^{-1} year^{-1}	N_2O kg N ha^{-1} year^{-1}	NEE g C m^{-2} year^{-1}	C budget g C m^{-2} year^{-1}	GWP t CO_2-eq ha^{-1} year^{-1}	Rate of change %
Wetland	Control	18.7±5.7	5.8±1.3	217.4±56.1	219.2±56.7	11.1±2.8	
	Rewetting	197.2±27.8	2.0±0.4	–129.4±38.5	–109.6±41.3	3.3±2.6	–70.4
	Control	2.3±0.9	1.6±0.7	299.2±31.4	299.4±31.4	11.8±1.5	
	MLTT	21.9±4.8	0.01±0.1	116.6±87.5	118.7±88.0	5.1±3.4	–56.8
	Control	5.4±1.4	ND	23.8±1.8	24.3±1.9	ND	
	NR	65.3±45.2	ND	–126.8±12.5	–120.2±17.0	ND	ND
	Control	198.0±0	2.7±1.2	147±0.0	166.8±0.0	13.7±0.5	
	Replanting	227.6±6.7	1.1±0.7	164.8±10.4	187.6±11.1	14.8±0.9	7.6
	Control	12.9±6.3	17.1±11.4	64.6±8.8	65.9±9.5	10.2±5.5	
	RR	55.9±8.5	4.0±1.3	–110.4±43.0	–104.9±43.8	–0.3±2.5	–102.9
Forest	P-Croplands	–1.3±0.3	3.7±0.9	–501.3	–501.4	–16.9±0.4	
	Forests	–2.5±0.2	1.4±0.3	–957.8	–957.1	–34.6±0.1	–105.4
	P-Grassland	–0.7±0.5	0.5±0.2	15.7±90.1	15.6±90.1	0.8±3.4	
	Forests	–1.4±0.3	1.4±0.4	–129.5±105.5	–129.7±105.5	–4.2±4.0	–651.8
Grassland	Control	–3.8±0.8	0.3±0.1	–587.9±119.1	–588.3±119.2	–21.6±4.4	
	RGD	–5.5±0.8	0.2±0.0	–1460.1±463.0	–1460.6±463.1	–53.7±17.0	–148.8
	Control	–2.6±0.4	0.6±0.2	–245.3±94.0	–245.6±94.1	–8.8±3.6	
	GE	–3.3±0.5	0.6±0.2	–703.0±188.4	–703.3±188.4	–25.6±7.0	–190.5
	Control	–2.5±0.8	1.2±0.5	96.9±363.6	96.6±363.7	4.0±13.6	
	AAR	–3.2±0.8	2.5±0.4	–255.4±402.9	–255.7±403.0	–8.4±15.0	–312.2
	Control	–0.8±0.4	2.3±0.7	10.3±30.1	10.2±30.2	1.3±1.4	
	C to R	–0.9±0.2	0.7±0.3	–75.8±20.1	–75.9±20.1	–2.5±0.9	–289.1

Table S3 Changes in comprehensive C budget and GWP in different grassland types. ND, no data; NEE, net ecosystem CO_2 exchange; C budget, the sum of NEE-C and CH_4-C; GWP, global warming potentials; TGM, Temperate steppe & meadow; AGM, Alpine steppe & meadow. Source data are provided as a Source Data file.

Grassland type	CH_4 kg C ha^{-1} year^{-1}	N_2O kg N ha^{-1} year^{-1}	NEE g C m^{-2} year^{-1}	C budget g C m^{-2} year^{-1}	GWP t CO_2-eq ha^{-1} year^{-1}	Rate of change %
Control	–2.6±0.4	0.5±0.3	–446.7±111.2	–447±111.3	–16.2±4.2	
TGM	–3.8±0.4	0.6±0.2	–1013.1±241.4	–1013.5±241.4	–37.0±8.9	–128.1
Control	–0.03±0.03	4.8±0.6	ND	ND	ND	
Prairie	0.0035±0.1	0.1±0.1	ND	ND	ND	ND
Control	–7.7±0	ND	61.0±36.3	60.2±36.3	ND	
Desert steppe	–11.4±0.4	ND	–144.9±112.8	–146.1±112.9	ND	ND
Control	–1.7±0.3	1.0±0.5	ND	ND	ND	
Artificial grassland	–1.7±0.2	0.5±0.1	ND	ND	ND	ND
Control	–1.9±0.9	0.6±0.3	–162.7±187.8	–162.8±187.9	–5.8±7.0	
AGW	–2.5±0.6	1.0±0.3	–726.6±311.3	–726.8±311.4	–26.3±11.6	–354.6
