# Supplementary material for: Meta-analysis shows the impacts of ecological restoration on greenhouse gas emissions
Source: Nat Commun. 2024 Mar 26;15:2668. doi: 10.1038/s41467-024-46991-5 (PMC10965928; doi:10.1038/s41467-024-46991-5)
Supplement: Supplementary file 3 — Description of Additional Supplementary Files [file 41467_2024_46991_MOESM3_ESM.pdf]

Description of Additional Supplementary Files

Supplementary Data 1 - All CH_4 data included in the presented meta-analysis.

Supplementary Data 2 - All N_2O data included in the presented meta-analysis.

Supplementary Data 3 - All CO_2 data included in the presented meta-analysis.

Supplementary Data 4 - All age data included in the presented meta-analysis.

Supplementary Data 5 - All references included in the presented meta-analysis.
