# Supplementary material for: Meta-analysis shows the impacts of ecological restoration on greenhouse gas emissions
Source: Nat Commun. 2024 Mar 26;15:2668. doi: 10.1038/s41467-024-46991-5 (PMC10965928; doi:10.1038/s41467-024-46991-5)
Supplement: Supplementary file 5 — Reporting Summary [file 41467_2024_46991_MOESM5_ESM.pdf]

nature portfolio

Corresponding author(s): Kerong Zhang

Last updated by author(s): Feb 23, 2024

Reporting Summary

Nature Portfolio wishes to improve the reproducibility of the work that we publish. This form provides structure for consistency and transparency in reporting. For further information on Nature Portfolio policies, see our Editorial Policies and the Editorial Policy Checklist.

Statistics

For all statistical analyses, confirm that the following items are present in the figure legend, table legend, main text, or Methods section.

n/a	Confirmed	
☐	☒	The exact sample size (n) for each experimental group/condition, given as a discrete number and unit of measurement
☐	☒	A statement on whether measurements were taken from distinct samples or whether the same sample was measured repeatedly
☐	☒	The statistical test(s) used AND whether they are one- or two-sided<br>*Only common tests should be described solely by name; describe more complex techniques in the Methods section.*
☐	☒	A description of all covariates tested
☐	☒	A description of any assumptions or corrections, such as tests of normality and adjustment for multiple comparisons
☐	☒	A full description of the statistical parameters including central tendency (e.g. means) or other basic estimates (e.g. regression coefficient) AND variation (e.g. standard deviation) or associated estimates of uncertainty (e.g. confidence intervals)
☐	☒	For null hypothesis testing, the test statistic (e.g. F, t, r) with confidence intervals, effect sizes, degrees of freedom and P value noted<br>*Give P values as exact values whenever suitable.*
☒	☐	For Bayesian analysis, information on the choice of priors and Markov chain Monte Carlo settings
☒	☐	For hierarchical and complex designs, identification of the appropriate level for tests and full reporting of outcomes
☒	☐	Estimates of effect sizes (e.g. Cohen's d, Pearson's r), indicating how they were calculated

Our web collection on statistics for biologists contains articles on many of the points above.

Software and code

Policy information about availability of computer code

Data collection	Peer-reviewed literature on greenhouse gas (GHG) emissions associated with ecological restoration were collected by searching the Google Scholar, Web of Science, and the China National Knowledge Infrastructure. Data in graphical figures and plots were extracted using Web Plot Digitizer (version 4.2).
Data analysis	MetaWin 3 software was used to calculate the overall effect size with a categorical random effects model. One-way analysis of variance (ANOVA) was used to test the differences in GHG fluxes (CH4, N2O, GPP, NEE and ER) and soil variables between the restored and paired control ecosystems using IBM SPSS Statistical Tool (Version 23.0; SPSS Inc.). Global distribution of the study sites was produced by ArcGIS 10.8 (Esri, West Redlands, CA, USA) software. There is no new R code for this paper.

For manuscripts utilizing custom algorithms or software that are central to the research but not yet described in published literature, software must be made available to editors and reviewers. We strongly encourage code deposition in a community repository (e.g. GitHub). See the Nature Portfolio guidelines for submitting code & software for further information.

Data

Policy information about availability of data

All manuscripts must include a data availability statement. This statement should provide the following information, where applicable:

- Accession codes, unique identifiers, or web links for publicly available datasets
- A description of any restrictions on data availability
- For clinical datasets or third party data, please ensure that the statement adheres to our policy

We systematically searched the peer-reviewed literature on greenhouse gas (GHG) emissions associated with ecological restoration from the Google Scholar, Web of Science, and the China National Knowledge Infrastructure. Our dataset included a paired restored-control samples sub-dataset and a chronosequence sub-dataset, which were compiled from 253 peer-reviewed articles published between December 1999 and June 2023. The paired sub-dataset included 679 paired measured cases, and the chronosequence sub-dataset included 1026 data points with restoration age (i.e., years since restoration). All data needed to evaluate the conclusions in the paper are present in the paper and/or Supplementary Data File. Source data are provided as a Source Data file.

Research involving human participants, their data, or biological material

Policy information about studies with human participants or human data. See also policy information about sex, gender (identity/presentation), and sexual orientation and race, ethnicity and racism.

Reporting on sex and gender	Not applicable. Our research does not involve human participants, their data, or biological material.
Reporting on race, ethnicity, or other socially relevant groupings	Not applicable. Our research does not involve human participants, their data, or biological material.
Population characteristics	Not applicable. Our research does not involve human participants, their data, or biological material.
Recruitment	Not applicable. Our research does not involve human participants, their data, or biological material.
Ethics oversight	Not applicable. Our research does not involve human participants, their data, or biological material.

Note that full information on the approval of the study protocol must also be provided in the manuscript.

Field-specific reporting

Please select the one below that is the best fit for your research. If you are not sure, read the appropriate sections before making your selection.

☐ Life sciences ☐ Behavioural & social sciences ☒ Ecological, evolutionary & environmental sciences

For a reference copy of the document with all sections, see nature.com/documents/nr-reporting-summary-flat.pdf

Ecological, evolutionary & environmental sciences study design

All studies must disclose on these points even when the disclosure is negative.

Study description	We conducted a global meta-analysis to examine the response of greenhouse gas (GHG) and environmental factors to ecological restoration, using a new global dataset compiled from 253 peer-reviewed articles. Hedges' d was used to evaluate the weighted response ratios (RRd) as it ranges from −∞ to +∞. MetaWin 3 software was used to calculate the overall effect size with a categorical random effects model43. Confidence intervals (95%; CIs) were calculated by bootstrapping (9,999 iterations).One-way analysis of variance (ANOVA) was used to test the differences in GHG fluxes (CH4, N2O, GPP, NEE and ER) and soil variables between the restored and paired control ecosystems.
Research sample	We searched the peer-reviewed literature on greenhouse gas (GHG) emissions associated with ecological restoration from the Google Scholar, Web of Science, and the China National Knowledge Infrastructure. Our dataset included a paired restored-control samples sub-dataset and a chronosequence sub-dataset, which were compiled from 253 peer-reviewed articles. The paired sub-dataset included 679 paired measured cases, and the chronosequence sub-dataset included 1289 data points with restoration age (i.e., years since restoration). The dataset is present in Supplementary Data File and the Source Data file.
Sampling strategy	We compiled a global dataset on greenhouse gas (GHG) associated with ecological restoration from peer-reviewed articles published between December 1999 and June 2023. We searched the literature on greenhouse gas (GHG) emissions associated with ecological restoration from the Google Scholar, Web of Science, and the China National Knowledge Infrastructure. Peer-reviewed studies were selected by the following criteria: (1) the selected experiments were conducted in the field from restored sites with paired control sites, or chronosequence sites; (2) each treatment was required to have at least three replicates; (3) the measurement covered an entire year or at least one growing season; (4) the selected studies reported at least one type of GHG. Finally, Our dataset included a paired restored-control samples sub-dataset and a chronosequence sub-dataset, which were compiled from 253 peer-reviewed articles. The paired sub-dataset included 679 paired measured cases, and the chronosequence sub-dataset included 1026 data points with restoration age (i.e., years since restoration). The sample sizes are sufficient because the dataset includes the main types of forest, grassland, and wetland restoration and covers the main regions of ecological restoration (please see the Figure 1 of the text).

	The dataset includes sufficient information for our analysis, such as the (1) GHG fluxes, including CH4, N2O, GPP, ER and net ecosystem CO2 exchange; (2) environmental factors, including longitude, latitude, mean annual air temperature (MAT), and mean annual precipitation (MAP); (3) restoration age, i.e., the years since restoration; (4) soil properties obtained from individual studies, including soil water table depth (WT), soil temperature (ST), and WFPS, Eh, BD, soil pH, SOC, TN, soil NH4+ and soil NO3−. The 253 peer-reviewed articles were listed in the Source Data file.
Data collection	Tiehu He and Kerong Zhang collected the data. We systematically searched the peer-reviewed literature from the Google Scholar, Web of Science, and the China National Knowledge Infrastructure using the following keywords: TS = (restoration * OR rehabilitation * OR revegetation * OR recovery * OR reconstruction * OR reclamation * OR restored * OR restoring * OR recovering *) AND TS = (methane * OR CH4 * or nitrous oxide * OR N2O * or carbon dioxide * OR CO2 * or greenhouse gas *) AND TS = (wetland * or forest * or grassland * or desert *). For each literature, we extracted the means, the number of replications, and standard deviations of the greenhouse gas (GHG), if reported. The authors of the 253 peer-reviewed literature were listed in the Supplementary Data File.
Timing and spatial scale	Articles published between December 1999 and June 2023. Global scale.
Data exclusions	No data were excluded from the analyses.
Reproducibility	The study is fully reproducible using the data and methods detailed in the manuscript.
Randomization	Not applicable - this is a meta-analysis based study.
Blinding	Not applicable - this is a meta-analysis based study.

Did the study involve field work? ☐ Yes ☒ No

Reporting for specific materials, systems and methods

We require information from authors about some types of materials, experimental systems and methods used in many studies. Here, indicate whether each material, system or method listed is relevant to your study. If you are not sure if a list item applies to your research, read the appropriate section before selecting a response.

Materials & experimental systems

n/a	Involved in the study
☒	☐ Antibodies
☒	☐ Eukaryotic cell lines
☒	☐ Palaeontology and archaeology
☒	☐ Animals and other organisms
☒	☐ Clinical data
☒	☐ Dual use research of concern
☒	☐ Plants

Methods

n/a	Involved in the study
☒	☐ ChIP-seq
☒	☐ Flow cytometry
☒	☐ MRI-based neuroimaging
